# A Novel Isolate with Deletion in *GP3* Gene of Porcine Reproductive and Respiratory Syndrome Virus from Mid-Eastern China

**DOI:** 10.1155/2014/306130

**Published:** 2014-02-12

**Authors:** Baochao Fan, Hai Wang, Juan Bai, Lili Zhang, Ping Jiang

**Affiliations:** Key Laboratory of Animal Diseases Diagnostic and Immunology, Ministry of Agriculture, College of Veterinary Medicine, Nanjing Agricultural University, Nanjing 210095, China

## Abstract

PRRSV strain SH1211 was isolated from the lung tissue of a piglet on a large-scale pig farm with approximately 30% morbidity and 50% mortality in mid-eastern China in 2012. The full-length genome of SH1211 was 15 313 nt in size, excluding the polyadenylated sequences, and shared 94.9% nucleotide sequence identity with the HP-PRRSV strain, JXA1. The GP2 and GP5 proteins of SH1211 shared only 91.5% and 85.1% amino acid sequence identities with those of the JXA1, respectively. A deletion at amino acid positions 68 and 69 was identified in the GP3 protein of SH1211, compared with the GP3 of Type-2 PRRSV isolates. A phylogenetic tree based on the nucleotide sequence of the complete genome showed that SH1211 is the most closely related to other HP-PRRSV strains isolated in China. However, phylogenetic analysis based on the GP2 and GP5 proteins showed that SH1211 is the most closely related to the QYYZ strain. A recombination analysis indicated that SH1211 might have been generated through recombination events between the JXA1 and QYYZ in which the GP2 and GP5 coding sequences were exchanged. Thus, SH1211 is a novel PRRSV strain with significant variation. Our analysis of SH1211 provides insight into the role of genetic variation in the antigenicity of PRRSVs in China.

## 1. Introduction

Porcine reproductive and respiratory syndrome virus (PRRSV), which causes great economic losses to the swine industry worldwide [1, 2], was first isolated in the Netherlands in 1990 [3] and was later identified in the USA [4]. The PRRSVs are divided into Type-1 (European, Lelystad prototype strain) and Type-2 (North American, VR-2332 prototype strain) genotypes, which vary in nucleotide sequence by approximately 60% [5, 6]. Along with equine arteritis virus, lactate dehydrogenase-elevating virus, and simian hemorrhagic fever virus, the PRRSVs are members of the order Nidovirales and family Arteriviridae [7, 8].

The PRRSV genome is 15 000 to 15 500 nt in length and consists of a 5′-untranslated region (UTR), at least 9 open reading frames (ORFs) that encode viral proteins, and a 3′-UTR [8, 9]. The ORFs 1a and 1b occupy the first two-thirds of the single-stranded, positive-sense RNA genome. These ORFs encode the ORF1ab replicase polyprotein, which is proteolytically cleaved into 13 small nonstructural proteins that are involved in virus replication and transcription [10, 11]. The PRRSV ORFs 2–7 encode a series of viral structural proteins that are associated with the virus envelope, including the GP2, E, GP3, GP4, GP5, M, and N proteins [8, 12–14]. A recent study identified ORF5a, which overlaps with the GP5-coding sequence and encodes a small hydrophobic protein [15, 16].

The PRRSV is characterized by extensive genetic variation, and a number of genetically/antigenically diverse strains have been identified [5, 17]. The coding region for the nonstructural protein 2 (nsp2) of PRRSV displays substantial genetic variation, including point mutations, insertions, and deletions [18–21]. The GP5 protein, a major component of the viral envelop, is thought to induce virus-neutralizing antibodies [22, 23] and displays the highest level of genetic variability among the PRRSV structural proteins [24, 25]. The GP3 protein is the second most heterogeneous structural protein, and the GP3 proteins of North American and European isolates share 54% to 60% amino acid sequence identity [26]. The GP3 protein has been the focus of studies of the genetic diversity, phylogenetic relationships, and molecular epidemiology of PRRSVs.

Although PRRSV isolates from all over the world cause similar symptoms and clinical features in pigs, previous studies have indicated that PRRSVs are antigenically, genetically, and pathologically heterogeneous [25, 27, 28]. In our current study, we isolated a novel PRRSV, the SH1211 strain, from a piglet on a large-scale pig farm with high morbidity and mortality in mideastern China in 2012. The genome of SH1211 was sequenced and analyzed to investigate the relationship between genetic variation and antigenicity among PRRSV isolates in China.

## 2. Materials and Methods

### 2.1. Clinical Samples

The lung tissue sample was obtained from a PRRSV-vaccinated piglet on a large-scale pig farm with approximately 30% morbidity and 50% mortality in Shanghai, China, in 2012. The piglet displayed clinical signs and symptoms that were typical of the porcine reproductive and respiratory syndrome (PRRS), including labored breathing, pyrexia, lethargy, and anorexia. The animal was diagnosed as PRRSV-positive using reverse transcription (RT) and polymerase chain reaction (PCR).

### 2.2. Virus Isolation

The lung tissue was homogenized and used to inoculate primary porcine alveolar macrophage (PAM) cells. The PAM cells were maintained in RPMI 1640 growth medium containing 10% heat-inactivated fetal bovine serum, 100 *μ*g/mL penicillin, and 100 U/mL streptomycin at 37°C in a 5% CO_2_ atmosphere until a cytopathic effect became visible. The infected cells were lysed using a freeze-thaw method and centrifuged at 2000 ×g for 10 min. The supernatant was stored at −70°C.

### 2.3. RNA Extraction and RT

Total RNA was extracted from the tissue homogenate using the RNeasy Mini Kit (Qiagen, Hilden, Germany) and suspended in nuclease-free water immediately before use. The RT procedure was performed using the SuperScript III First-Strand Synthesis Kit (Invitrogen, Carlsbad, CA, USA). Samples containing 8 *μ*L of total RNA, 1 *μ*L of 50 *μ*M oligo (dT)_20_ primer, and 1 *μ*L of 10 mM dNTP mix were incubated at 65°C for 5 min for primer annealment and placed on ice for at least 1 min immediately afterward. For first-strand synthesis, 2 *μ*L of 10× RT buffer, 4 *μ*L of 25 mM MgCl_2_, 2 *μ*L of 0.1 M DTT, 1 *μ*L of 40 U/*μ*L RNaseOUT (Invitrogen), and 1 *μ*L of 200 U/*μ*L Superscript III reverse transcriptase were added to each sample, and the samples were incubated at 50°C for 50 min. The RT reaction was terminated by incubation at 85°C for 5 min, and the samples were placed on ice until complementary DNA (cDNA) synthesis was performed.

### 2.4. Primer Design and cDNA Synthesis

The ORF5 nucleotide sequence of SH1211 was compared with that of other PRRSV isolates available in GenBank database (NCBI) to identify similarities, upon which primers were designed. The SH1211 genome was divided into 7 overlapping fragments for amplification, and the 5′ and 3′ termini of the genomic sequence were synthesized using rapid amplification of the cDNA ends (RACE). The sequences of the primers used for the whole genome and the RACE procedure are provided in Table 1. The overlapping fragments were amplified by PCR using the Phanta Super Fidelity DNA Polymerase (Vazyme, China), and the thermal cycling was performed at 95°C for 3 min, followed by 35 cycles of 95°C for 10 s, 52°C to 56°C for 30 s, and 72°C for 2 min, with a final extension at 72°C for 10 min. The amplified PCR products were analyzed using agarose gel electrophoresis.

### 2.5. Genome Cloning and Sequencing

The PCR products were purified from the agarose gel using the AxyPrep DNA Gel Extraction Kit (Axygen, China) and cloned into the pEASY-Blunt Zero vector (Trans, China). Three clones were sequenced by a commercial service provider (Invitrogen, Shanghai, China).

### 2.6. Nucleotide and Amino Acid Sequence Analyses

The overlapping sequences of the PCR products were combined to obtain the full-length genomic sequence of the SH1211 strain. A nucleotide BLASTn analysis was used to compare the sequences of the SH1211 genes with those of the reference strains of PRRSV in the GenBank database (Table 2). The sequence alignments were generated using the Clustal W program. Phylogenetic trees of the full-length genomic, GP2, and GP5 nucleotide sequences were generated using the distance-based Neighbor-Joining method in the MEGA, version 5.05, software. The bootstrap values were calculated based on 1000 replicates, and the evolutionary distances were computed using the Jukes-Cantor method. The amino acid (AA) sequence comparisons were obtained using the BioEdit software.

### 2.7. Recombination Analysis

Multiple sequence alignments between SH1211 and the other PRRSV strains were performed using the Clustal X, version 1.83, program. The Recombination Detection Program (RDP4), version 4.1.3, was used to estimate the potential recombination events [29]. The GENECONV method was used to search for putative breakpoints [30].

## 3. Results

### 3.1. Comparison of Full-Length Genomic Sequences of SH1211 and Other PRRSV Strains

The sequence analysis showed that the full-length genomic sequence of SH1211 was 15 313 nucleotides in length, excluding the polyadenylated sequences, and included the following genes and UTRs: 5′ UTR (188 nt), *Rep* (11 792 nt), *GP2* (771 nt), *GP3* (759 nt), *GP4* (537 nt), *GP5* (603 nt), *M* (525 nt), *N* (372 nt), and 3′ UTR (150 nt). The nucleotide sequence of the full-length SH1211 genome was compared with those of the other PRRSV isolates, including two North American strains (MN184C and VR-2332), one European strain (LV), and six Chinese strains (CH-1a, SY0608, QYYZ, JXA1, WUH4, and HB-1(sh)/2002) (Table 3).

**Figure 1:**
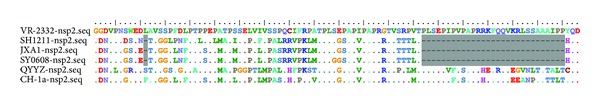
Alignment of partial nsp2 amino acid sequence of SH1211 with several representative PRRSV strains. The amino acid deletions are shown with grey bar.

The SH1211 strain shared a higher level of nucleotide sequence identity with the Type-2 PRRSV strains (81.8% to 95.1%), compared to that shared with the Type-1 PRRSV strain, LV (58.0%). The highest levels of shared nucleotide sequence identity (94.9% to 95.1%) were observed between the SH1211 strain and the WUH4, JXA1, and SY0608 strains, which had previously caused epidemics in China. Moreover, the 950-AA sequence of the protein produced from the *Nsp2* gene (2850 nt) of the SH1211 strain contained 30 noncontiguous AA deletions, relative to that of the *Nsp2* gene of the VR-2332 strain (Figure 1). These results indicated that the SH1211 strain is highly similar to a group of highly pathogenic (HP) strains of PRRSV previously isolated in China.

The nucleotide lengths of ORF1a and ORF1b of SH1211 were 7422 nt and 4383 nt, respectively. The ORF1a and ORF1b of SH1211 shared nucleotide sequence identities of 96.0% and 94.9% with those of the JXA1 strain, respectively, whereas it shared 53.2% and 63.0% with those of the LV strain, respectively. The predicted AA sequences of the proteins of SH1211 shared 97.2% to 99.4% identity with those of the JXA1 strain, except for the Nsp1*β*, Nsp2, and Nsp12 proteins, which were only 92.6%, 94.2%, and 93.4%, respectively. The Nsp1*β* protein of SH1211 shared 91.6% AA sequence identity with that of the SY0608 strain. These data indicated that a high level of variation occurred in the Nsp1*β* protein of the SH1211 strain.

The comparisons of the sequences of ORFs 2 through 7 showed that the SH1211 strain shared 84.4% to 97.5% nucleotide sequence identity with the Type-2 PRRSV strains. The ORF2 and ORF5 of SH1211 exhibited the highest levels of variability, compared with the various HP-PRRSV strains, with 91.3% and 84.4% nucleotide sequence identities shared with the JXA1 strain, respectively. The levels of nucleotide sequence identity shared between the SH1211 and JXA1 strains for the other ORFs ranged from 94.9% to 97.5%. The ORF2 and ORF5 of SH1211 shared nucleotide sequence identities of 92.9% and 92.8%, respectively, with the QYYZ strain, and the predicted proteins for ORFs 2 and 5 of SH1211 shared AA sequence identities of 93.3% and 94.0%, respectively, with those of the QYYZ strain.

### 3.2. Phylogenetic Analyses

**Figure 2:**
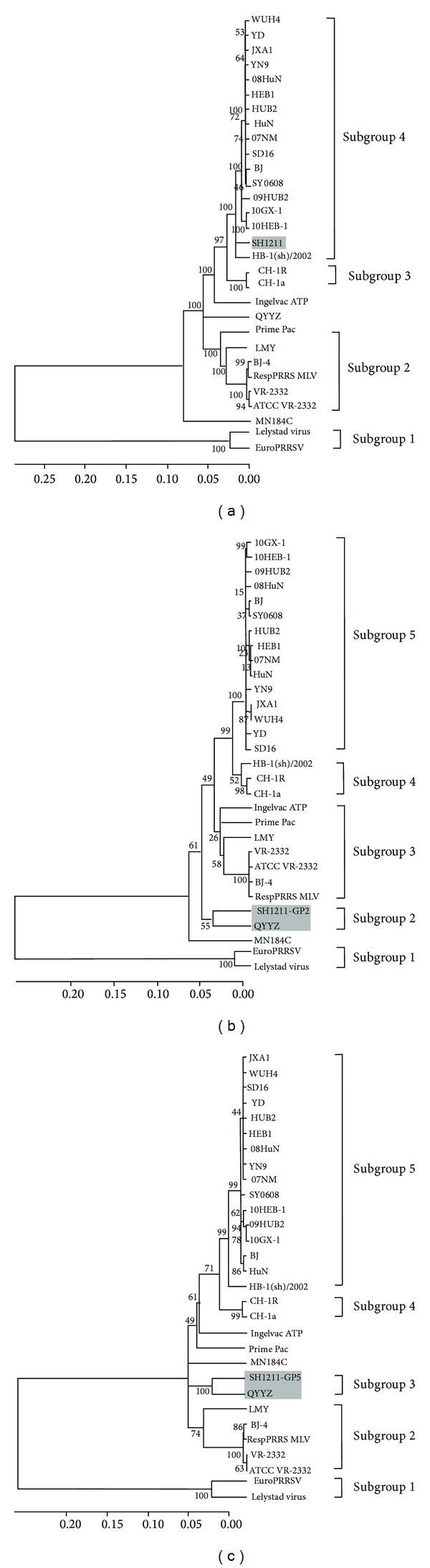
Phylogenetic analysis based on nucleotide sequences of the full-length genome (a), ORF2 (b), and ORF5 (c) of 30 fully sequenced PRRSV isolates. The evolutionary history was inferred using the Neighbor-Joining method. The percentage of replicate trees in which the associated taxa clustered together in the bootstrap test (1000 replicates) was shown next to the branches. The evolutionary distances were computed using the Jukes-Cantor method and were in the units of the number of base substitutions per site. Evolutionary analyses were conducted in MEGA5.

Phylogenetic trees were produced based on the nucleotide sequences of the full-length genome, ORF2, and ORF5 of SH1211 and the various PRRSVs (Figures 2(a)–2(c), resp.). The sum of the branch lengths for the optimal trees for the full-length genome, ORF2, and ORF5 sequences were 1.057, 0.843, and 1.070, respectively. Based on the full-length genome sequence, the PRRSV strains were divided into subgroups 1–4. The SH1211 strain was assigned to subgroup 4, which consisted of 17 PRRSV strains collected in China from 2002 to 2012. However, the SH1211 strain formed a minor branch that was separate from that containing the other subgroup-4 strains. The ORF2 phylogenetic tree revealed five different subgroups. The SH1211 and QYYZ strains formed subgroup 2, which separated from subgroup 5 by a significant evolutionary distance. The ORF5 phylogenetic tree revealed similar relationships among the various PRRSV strains, with SH1211 and QYYZ forming subgroup 3. These data indicated that significant variation occurred in ORFs 2 and 5 of SH1211 and QYYZ, compared with the other PRRSVs.

### 3.3. Recombination Analysis

**Figure 3:**
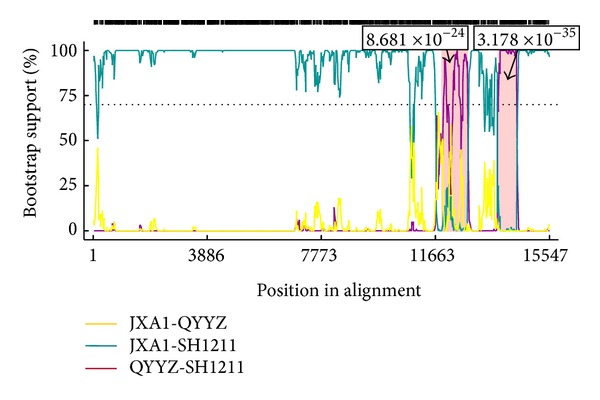
The recombinant event of SH1211 was analyzed by the BootScan method in software RDP4. The *x*-axis shows the position in alignment, and the *y*-axis shows the bootstrap support (%). Major parent plot (blue line) and minor parent plot (purple line) cross two areas (pink regions), which represent the recombinant regions, and the numbers of approximate *P* values locate at the top of pink regions, respectively. The bootstrap cut-off is 70%.

As shown in Figures 2(b) and 2(c), based on the ORF2 and ORF5 nucleotide sequences, the QYYZ strain displayed the closest phylogenetic relationship with the SH1211 strain. The SH1211 genome was subjected to a recombination analysis, and the potential breakpoints with optimal *P* values based on the *χ* ^2^ analysis were identified using the GENECONV method. Four potential recombination breakpoints were identified at nucleotide positions 11 697, 12 768, 13 819, and 14 472 (Figure 3), and the approximate *P* values for the fragments corresponding to nucleotide positions 11 697–12 768 and 13 819–14 472 were 8.681 × 10^−24^ and 3.178 × 10^−35^, respectively. The *GP2 *and *GP5 *genes of the SH1211 strain were located within these two recombinant regions. These data suggest that the JXA1 and QYYZ strains recombined to form the SH1211 strain.

### 3.4. Sequence Analyses of the UTRs

**Figure 4:**
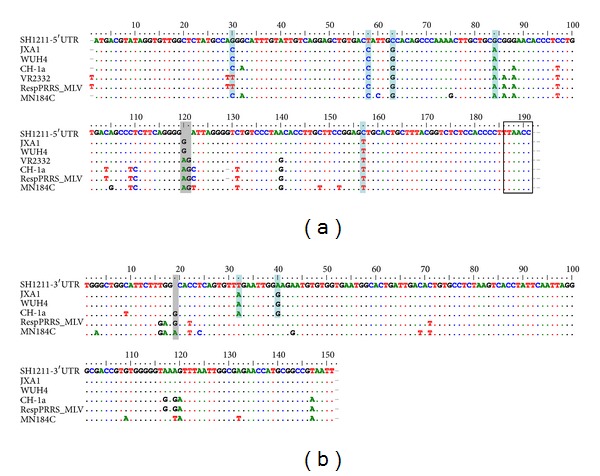
Alignment of nucleotide sequences of 5′UTR (a) and 3′UTR (b) of SH1211 with several representative PRRSV strains. The nucleotide deletions and the key nucleotide mutations within the different strains are shown with gray bar and blue bar, respectively. The transcription regulatory sequence (TRS) UUAACC is indicated by a black box.

The 188-nt 5′ UTR of SH1211 shared a higher level of sequence identity (96.8%) with JXA1 than that shared with LV (53.3%). The nucleotide alignments revealed a deletion in the 5′ UTR of SH1211 at nucleotide position 119, relative to the JXA1 and WUH4 strains, and positions 119 and 120, relative to the CH-1a strain. Point mutations were identified in the 5′ UTR of SH1211 at positions 29 (C→G), 57 (C→U), 62 (G→C), 83 (A→G), and 156 (U→C), relative to the 5′ UTR of the JXA1 strain. Furthermore, the transcription regulatory sequence, UUAACC, was identified at the 3′ boundary of the 5′ UTR in the SH1211 genome. The 3′ UTR of the SH1211 genome shared 84.9% to 98.0% sequence identities with the Type-2 isolates. The 3′ UTR of the SH1211 genome had a deletion at nucleotide position 19, relative to that of the CH-1a strain (Figure 4(b)), and two point mutations at positions 32 (A→U) and 40 (G→A), relative to the 3′-UTRs of the JXA1 and WUH4 strains.

### 3.5. Amino Acid Analysis of *GP3*

**Figure 5:**
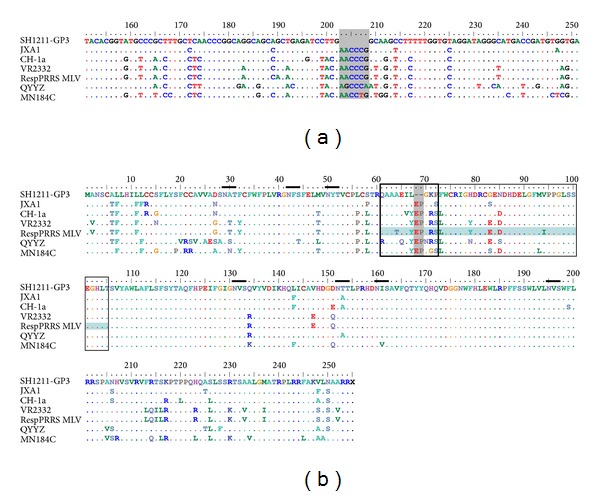
Alignment of partial GP3 nucleotide sequence (a) and whole GP3 amino acid sequences (b) of SH1211 with several representative PRRSV strains. The nucleotide and amino acid deletions are shown with grey bar; the key amino acid mutations are shown with blue bar. The antigenic region Q^61^AARQRLEPGRN^72^ and the immunoreactive area (AA positions 61–105) are indicated by black boxes. The short lines represent N-glycosylation sites.

As shown in Figure 5, the *GP3* gene of SH1211 had a 6-nt deletion at positions 203–208 (corresponding to AA positions 67 and 68), relative to the *GP3* gene of the Type-2 PRRSV strains. A two-AA deletion (E^68^ and P^69^) was identified in the antigenic region of the predicted GP3 polypeptide, relative to that of the other PRRSVs examined. The AA substitutions, T^64^→A^64^, Y^67^→L^67^, R^71^→K^69^, S^72^→P^70^, L^73^→F^71^, Y^79^→H^77^, E^83^→G^81^, D^85^→N^83^, and I^94^→V^92^, were identified in the main antigenic region of the predicted GP3 polypeptide of SH1211, relative to the GP3 AA sequence of the RespPRRS MLV strain. In addition, the predicted GP3 polypeptide of SH1211 shared seven potential N-glycosylation sites, N^29^, N^42^, N^50^, N^129^, N^150^, N^158^, and N^193^, with the Type-2 PRRSV strains [31].

### 3.6. Amino Acid Analysis of GP5

**Figure 6:**
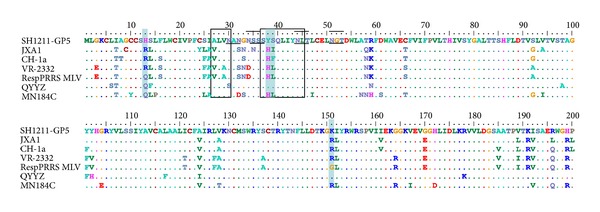
Alignment of whole GP5 amino acid sequence of SH1211 with several representative PRRSV strains. The key amino acid mutations within the different strains are shown with blue bar. The decoy epitope and the primary neutralizing epitope (PNE) are indicated by boxes. The short lines represent N-glycosylation sites.

Most of the AA substitutions identified in the predicted GP5 polypeptide of SH1211 were located within residues 1 to 31 in the N-terminal signal sequence, residues 32 to 60 in the hypervariable region of the ectodomain, and residues 189 to 200 in the C-terminal endodomain (Figure 6). Two AA substitutions, H^38^→Y^38^ and F/L^39^→S^39^, were identified in the primary neutralizing epitope (PNE), S^37^H(F/L)QLIYNL [32], of the SH1211 strain, compared with that in the VR-2332 strain and its attenuated vaccine derivative, RespPRRS MLV. No AA mutations were found in the decoy epitope of the predicted GP5 protein of SH1211. Residues 13 and 151 in the GP5 proteins of PRRSVs have been shown to be associated with the virulence [6]. However, the AA substitutions, H^13^ and K^151^, were identified in the predicted GP5 protein of the SH1211 strain. To gain further insight into the genetic evolution of SH1211, we also analyzed variation in potential N-glycosylation sites. The predicted GP5 protein of the SH1211 strain shared three N-glycosylation sites with the CH-1a strain at AA positions 34, 44, and 51, and the deletion of two N-glycosylation sites at positions 31 and 35 was identified, relative to those in the GP5 protein of the JXA1 strain.

## 4. Discussion

The PRRS continues to be a serious threat, causing a significant economic impact on the swine industry worldwide. Although commercial vaccines against PRRSV are available, traditional control strategies and conventional vaccines have failed to provide sustainable disease control. Surveillance of the recently emerged strains has become necessary because of the considerable genetic and antigenic diversity identified in these HP-PRRSV isolates. In our current study, a novel variant PRRSV strain was isolated from a piglet in a PRRSV-vaccinated pig herd with high morbidity and mortality in mid-eastern China.

The GP3 protein of PRRSVs is a minor structural protein. The 254-AA GP3 protein is encoded by ORF3 and has a molecular mass of approximately 42 kDa [33, 34]. The GP3 protein is one of the most variable structural proteins among the PRRSVs, with only 54% to 60% AA sequence identity shared between the Type-1 and Type-2 strains [24, 35]. In the SH1211 strain, the length of ORF3 was 759 nt, and it had a six-nucleotide deletion at positions 203–208, which correspond to AA positions 67 and 68, relative to the Type-2 PRRSV strains. In the GP3 protein, four consecutive overlapping epitopes, corresponding to AA positions 61–105, were shown to be strongly immunoreactive to 85% to 100% of the anti-PRRSV sera tested [36]. These epitopes are located in the most hydrophilic region within the GP3 protein, and are considered to comprise an important immunoreactive region of the North American strains of PRRSV [36].

The AA sequence, Q^61^AARQRLEPGRN^72^, of the antigenic region has been shown to be the target of virus-neutralizing antibodies [37]. It is notable that a two-AA deletion (E^68^ and P^69^) occurs in the antigenic region of SH1211. Multiple AA substitutions were also identified in the primary antigen region of the GP3 protein of the SH1211 strain, relative to that of the RespPRRS MLV attenuated vaccine strain. A recent study demonstrated that an N-glycan moiety in GP3 is responsible for glycan shield interference, which can influence the ability of the host to produce neutralizing antibodies [38]. However, the two-AA deletion in the GP3 of the SH1211 strain did not affect any of the seven potential N-glycosylation sites identified. Future studies of the sequence diversity in the SH1211 strain are warranted to determine its relationship with immunogenicity.

The envelope protein, GP5, is the most variable protein of the PRRSVs, with only 51% to 55% sequence identity shared between the Type-1 and Type-2 strains [28, 35]. Hypervariability in GP5 is likely responsible for the low level of immunological cross-reactivity observed between the PRRSVs [17]. Thus, GP5 has become the focus of analyses of the genetic diversity of PRRSVs [27, 39, 40]. Our analysis of the GP5 of the SH1211 strain revealed significant variation, with only 84.4% nucleotide identity shared between the ORF5 of SH1211 and that of the representative Chinese HP-PRRSV strain, JXA1. The PNE is also an important domain of GP5 with regard to virus neutralization, and the H^38^(L/F)^39^ residues in this domain are considered to be critical to the immunogenicity of this epitope [32, 41]. The PNE of the SH1211 strain had the H^38^→Y^38^ and F/L^39^→S^39^ AA substitutions in the PNE, which probably contributed to the ability of the isolate to escape neutralizing antibodies induced by PRRSV vaccines used in China, including the attenuated RespPRRS/Repro vaccine strain, the CH-1R vaccine strain (attenuated vaccine strain derived from CH-1a), and the attenuated live vaccine strain derived from JXA1.

The decoy epitope of GP5 is comprised of (A/V)^27^LVN near the PNE, and may delay the production of virus-neutralizing antibodies [32]. However, no AA mutations were found in the decoy epitope of the GP5 of the SH1211 strain. A recent study identified two identical signal peptide cleavage sites in the GP5 of PRRSVs, which results in a mixture of GP5 proteins in virus particles, one with and one without the decoy epitope [42]. By comparing the cleavage of wild-type GP5 to that of mutant viruses in which cleavage site 1 or 2 was blocked, the majority of GP5 was found to be cleaved at site 2, which deleted the decoy epitope. Thus, other factors may play vital roles in delaying the production of virus-neutralizing antibodies [42]. Residues 13 and 151 of GP5 are associated with the virulence of PRRSVs. The R^13^→Q^13^ and R^151^→G^151^ AA substitutions in VR-2332 resulted in the attenuation of the RespPRRS MLV vaccine strain [6]. The GP5 of the SH1211 strain contained H^13^ and K^151^ at these key residues. Future studies of the virulence of the SH1211 strain are warranted to determine the influence of these mutations in GP5 on pathogenicity.

The number of the potential N-glycosylation sites apparently increased in the Chinese isolates over time [43]. Additional N-glycosylation sites may lead to an increase in the number of the N-linked glycans on the envelope surface, which provide a barrier to antibodies. Variation in the glycan shield, therefore, presents a possible mechanism for reduced antigenicity [44]. In our current study, the GP5 of the SH1211 strain had N-glycosylation sites at positions 34, 44, and 51 only, which are identical to those in the classical PRRSV strain, CH-1a. However, the high morbidity and mortality in the SH1211-infected herd indicate that other important factors contribute to the virulence of the virus. A recent study showed that an ORF5a wobble position in PRRSVs is used as a highly selective codon usage mechanism that conserves the RQ-motif in the ORF5a protein, despite significant selective pressure on the GP5 N-linked glycosylation motifs [45]. Future studies of the relationship between the variations in ORF5a protein and the N-glycosylation sites in GP5 of the SH1211 strain are warranted.

The ORF2 and ORF5 of the SH1211 strain shared high levels of sequence identity with the QYYZ strain. However, the remaining ORFs of SH1211 shared higher levels of sequence identity with the JXA1 strain. In the phylogenetic trees based on the ORF2 and ORF5 nucleotide sequences, the SH1211 strain clustered with the QYYZ strain, whereas the phylogenetic tree based on the complete genome sequence showed substantially different relationships between the SH1211 strain and other PRRSVs. Therefore, we analyzed possible recombination events in the SH1211 strain, and the results indicated that the nucleotide sequences from positions 11 697–12 768 and 13 819–14 472 of SH1211 were derived from the QYYZ strain, while the remaining segments were from the HP-PRRSV strain, JXA1. The two recombinant QYYZ-like regions contained the ORFs encoding the GP2 and GP5 proteins. Thus, these recombination events might have contributed to the virulence of the SH1211 strain. Because the sequence identities shared between the SH1211 and QYYZ strains for the two recombinant regions were 92.9% and 92.8%, the statistical significance of the recombination breakpoints indicates that the SH1211 strain is a recombinant virus derived from the HP-PRRSV strain, JXA1, and the wild-type PRRSV strain, QYYZ.

The ORF1a and ORF1b of PRRSVs encode the long nonstructural polyproteins, PP1a and PP1ab, with the expression of the latter depending on a ribosomal frame-shift signal in the ORF1a/ORF1b overlap region [46]. The PP1a and PP1ab polyproteins are cleaved by viral proteases to release 14 nonstructural proteins, which include four proteases (Nsp1*α*, Nsp1*β*, Nsp2, and Nsp4), an RNA-dependent RNA polymerase (Nsp9), a helicase (Nsp10), and an endonuclease (Nsp11) [46–48]. The Nsp1*β* protein functions in the inhibition of interferon (IFN)-*β* transcription [49], suppresses both IRF3- and NF-*κ*B-mediated IFN gene expression [49–51], and interferes with IFN-induced JAK-STAT signaling [50, 52]. A recent study indicated that the Nsp1*β* blocks the nuclear translocation of interferon-stimulated gene factor 3 (ISGF3) by inducing the degradation of karyopherin-*α*1 and that V^19^ in the Nsp1*β* protein correlates with the inhibition of ISGF3 translocation [53].

Among the nonstructural proteins of the SH1211 strain of PRRSV, the Nsp1*β* shared the lowest level of sequence identity (92.6%) with JXA1. Whether the high frequency of mutations in the Nsp1*β* protein contributes to the suppression of the host innate immune response to the SH1211 strain remains unclear. In addition, the 5′ and 3′ UTRs of PRRSVs have been shown to be important regulatory elements [54, 55]. Future studies are warranted to determine whether the nucleotides deletions in the 5′ and 3′ UTRs affect the replication, transcription, and virulence of the SH1211 strain.

## 5. Conclusions

The SH1211 strain represents a recently emerging virus with a genome structure that is typical of PRRSVs with unique genetic variation, including a deletion in the *GP3* gene and apparent recombinations involving the coding sequences for the GP2 and GP5 proteins. Our sequence analysis of the SH1211 strain provides insight into the role of genetic variation in the antigenicity of PRRSVs in China. Future studies of the immunogenicity and pathogenicity of the SH1211 strain are warranted to identify the mechanisms underlying the contribution of genetic variation in PRRSVs to host-pathogen interactions.

## Figures and Tables

**Table 1:** Primers used for RT-PCR and RACE amplification for SH1211.

Primers name	Sequence	Genome position in JXA1
A1-400	5′-AGCACTCGACAGTG-3′	400
S1-412	5′-CGATTGCACGAATGACTA-3′	412
A2-381	5′-TGCACGTGGCAACGTC-3′	381
S2-437	5′-CGATTGCACGAATGACTA-3′	437
RT488	5′-CGCACCATTCTTTGTTGA-3′	488
F-322	5′-GGGGTGCTGGGTCTATT-3′	322
R-2352	5′-ATAGCAATTGGACAGTGAGAAG-3′	2352
F-2244	5′-CGAACAACCTCACGTCAACTCA-3′	2244
R-4588	5′-GTAATACCCGCAAGCACTTTAC-3′	4588
F-4512	5′-GAAGAGGATTACGGCTAGAACT-3′	4512
R-6878	5′-TTATCAACCTGTACCAACTGAG-3′	6878
F-6781	5′-TCTGCGTCCAACATGAGGAATG-3′	6781
R-9137	5′-CAGCACAAGGTCGTCCGAATAG-3′	9137
F-9059	5′-GTTTCTGCAAGACCAGCTAAAG-3′	9059
R-11471	5′-CTCCTTGAAGTCCAACATCACT-3′	11471
F-11387	5′-GGATGTGTACCTCCCAGACCTT-3′	11387
R-13812	5′-TATGAGAGCTGTTGTTGTTGCT-3′	13812
F-13683	5′-TTTGAATGTTCAAGTATGTTGG-3′	13683
R-14755	5′-ACCCAACACGAGGCTTTTCAAC-3′	14755
3′RACE	5′-ACATTCGTGCACTTTGAGAG-3′	14465

**Table 2:** Representative PRRSV Strains used in this study.

No.	Name	Country	Year	Accession number
1	CH-1a	China	1996	AY032626
2	BJ-4	China	2000	AF331831
3	HB-1(sh)/2002	China	2002	AY150312
4	HEB1	China	2006	EF112447
5	JXA1	China	2006	EF112445
6	HUB2	China	2006	EF112446
7	HuN	China	2007	EF517962
8	SY0608	China	2007	EU144079
9	07NM	China	2007	FJ393456
10	BJ	China	2007	EU825723
11	CH-1R	China	2008	EU807840
12	YN9	China	2008	GU232738
13	08HuN	China	2008	GU169411
14	09HUB2	China	2009	JF268683
15	10GX-1	China	2010	JQ663558
16	10HEB-1	China	2010	JQ663551
17	WUH4	China	2011	JQ326271
18	QYYZ	China	2011	JQ308798
19	YD	China	2012	JF748717
20	SD16	China	2012	JX087437
21	SH1211	China	2012	KF678434
22	LMY	South Korea	2002	DQ473474
23	VR-2332	USA	1992	AY150564
24	EuroPRRSV	USA	1999	AY366525
25	MN184C	USA	2007	EF488739
26	Ingelvac ATP	USA		DQ988080
27	Prime Pac	USA		DQ779791
28	RespPRRS MLV	USA	1994	AF066183
29	ATCC VR-2332	USA	1990	U87392
30	Lelystad virus	Europe	1991	M96262

**Table 3:** Nucleotide and deduced amino acid identities of SH1211 compared with those of WUH4, QYYZ, HB-1(sh)/2002, JXA1, SY0608, MN184C, VR-2332, CH-1a, and LV (%).

SH1211%	Identity to SH1211								
	WUH4	QYYZ	HB-1(sh)/2002	JXA1	SY0608	MN184C	VR-2332	CH-1a	LV
Nucleotides (length)									
5′UTR (188)	96.8	92.6	93.6	96.8	96.3	88.9	91.0	94.2	53.3
ORF1a (7422)	96.1	82.0	93.0	96.0	96.1	76.5	84.7	90.8	53.2
ORF1b (4383)	94.8	89.0	93.9	94.9	94.9	86.4	89.9	92.8	63.0
ORF2 (771)	91.3	92.9	92.2	91.3	91.4	87.1	91.1	92.7	63.8
ORF3 (759)	94.9	88.7	92.9	95.2	95.6	84.7	87.3	92.9	61.5
ORF4 (537)	96.0	93.8	94.4	95.3	96.8	86.2	89.1	94.4	64.3
ORF5 (603)	83.9	92.8	84.4	84.4	84.4	85.0	84.5	85.5	59.0
ORF6 (525)	97.5	90.0	97.1	97.5	97.5	90.6	94.6	95.2	68.5
ORF7 (372)	96.7	87.9	96.2	97.0	96.7	90.0	92.2	94.8	61.5
3′UTR (150)	98.0	84.9	94.7	98.0	97.3	90.7	54.9	94.7	55.9
Complete (15,313)	95.0	86.0	92.9	94.9	95.1	81.8	87.4	91.7	58.0

Amino acid (length)									
nsp1a (180)	94.4	96.1	97.7	99.4	99.4	94.4	95.0	96.6	65.0
nsp1b (203)	92.6	81.2	90.6	92.6	91.6	75.8	80.2	84.2	39.7
nsp2 (950)	94.1	74.6	89.2	94.2	94.1	62.3	76.7	84.8	33.8
nsp3 (446)	97.6	90.0	96.9	98.0	98.0	93.0	95.3	98.0	56.1
nsp4 (204)	98.0	91.1	97.5	98.0	98.0	93.1	94.1	95.5	62.7
nsp5 (170)	98.2	84.9	98.2	98.2	97.6	85.8	92.3	94.7	71.1
nsp6 (16)	100	100	100	100	100	93.7	93.7	100	75.0
nsp7 (259)	98.0	94.5	95.7	98.4	98.4	84.9	88.4	95.3	43.7
nsp8 (45)	97.7	97.7	95.5	97.7	97.7	95.5	97.7	97.7	66.6
nsp9 (643)	98.3	98.3	96.7	97.3	97.7	96.0	97.8	98.1	75.9
nsp10 (441)	97.2	96.3	97.1	97.2	97.1	93.9	94.8	96.8	65.0
nsp11 (223)	98.6	95.5	96.8	98.2	98.2	94.6	96.4	97.7	76.3
nsp12 (153)	93.4	92.1	93.4	93.4	94.3	94.7	91.5	93.4	39.2
GP2 (257)	91.0	93.3	90.6	91.0	91.0	84.3	88.6	91.7	60.1
E (74)	90.5	95.9	90.5	90.5	90.5	93.2	93.2	90.5	70.2
GP3 (253)	92.5	85.4	89.3	93.3	94.0	85.0	85.8	89.3	53.0
GP4 (179)	95.5	94.3	97.1	94.3	97.7	89.3	89.3	97.1	69.3
GP5 (201)	84.5	94.0	84.5	85.0	84.5	85.0	82.5	85.5	52.6
M (175)	98.8	96.5	98.8	98.8	98.8	96.5	97.7	97.1	79.3
N (124)	97.5	91.0	95.9	97.5	96.7	91.0	93.4	94.3	58.0
